# Supplementary material for: Comparative Proteomic and Metabolomic Analysis of Human Osteoblasts, Differentiated from Dental Pulp Stem Cells, Hinted Crucial Signaling Pathways Promoting Osteogenesis
Source: Int J Mol Sci. 2021 Jul 24;22(15):7908. doi: 10.3390/ijms22157908 (PMC8347416; doi:10.3390/ijms22157908)
Supplement: Supplementary file 1 [file ijms-22-07908-s001.zip › Appendix A, Supplementary figures, Figure S1-S3.pdf]

Comparative proteomic and metabolomic analysis of human osteoblasts, differentiated from dental pulp stem cells, hinted crucial signaling pathways promoting osteogenesis

Slavomíra Nováková[1*#], Maksym Danchenko[2#], Terézia Okajčeková[1], Eva Baranovičová[1], Andrej Kováč[3], Marián Grendár[1], Gábor Beke[4], Janka Pálešová[1], Ján Strnádel[1], Mária Janíčková[5], Erika Halašová[1,6] and Henrieta Škovierová[1*]

[1]Biomedical Centre Martin, Jessenius Faculty of Medicine in Martin, Comenius University in Bratislava (JFM CU), Malá Hora 4C; 036 01 Martin, Slovak Republic

[2]Plant Science and Biodiversity Center, Slovak Academy of Sciences, Dúbravská cesta 9, 845 23 Bratislava, Slovak Republic

[3]Institute of Neuroimmunology, Slovak Academy of Sciences; Dúbravská cesta 9, 845 10 Bratislava, Slovak Republic

[4]Institute of Molecular Biology, Slovak Academy of Sciences; Dúbravská cesta 21, 845 51 Bratislava, Slovak Republic

[5]Department of Stomatology and Maxillofacial Surgery, University Hospital in Martin and JFM CU, Kollárova 2, 036 01 Martin, Slovak Republic

[6]Department of Medical Biology, Jessenius Faculty of Medicine in Martin, Comenius University in Bratislava (JFM CU), Malá Hora 4, 036 01 Martin, Slovak Republic

*Correspondence: henrieta.skovierova@uniba.sk; Tel.: +421 43 2633 904 (H.S.)
slavomira.novakova@uniba.sk; Tel.: +421 43 2633 904 (S.N.)

# Both authors contributed equally to this work

Table of content

Supplementary figure S1. Morphological changes during 24 days in undifferentiated (control) (A) and differentiated cells (B). Magnification 100 x.

Supplementary figure S2. Principal component analysis: A—proteins, B—metabolites

Supplementary figure S3. MetaboAnalyst pathway analysis. Column chart show functional categorization of differentially abundant metabolites into metabolic pathways.

A

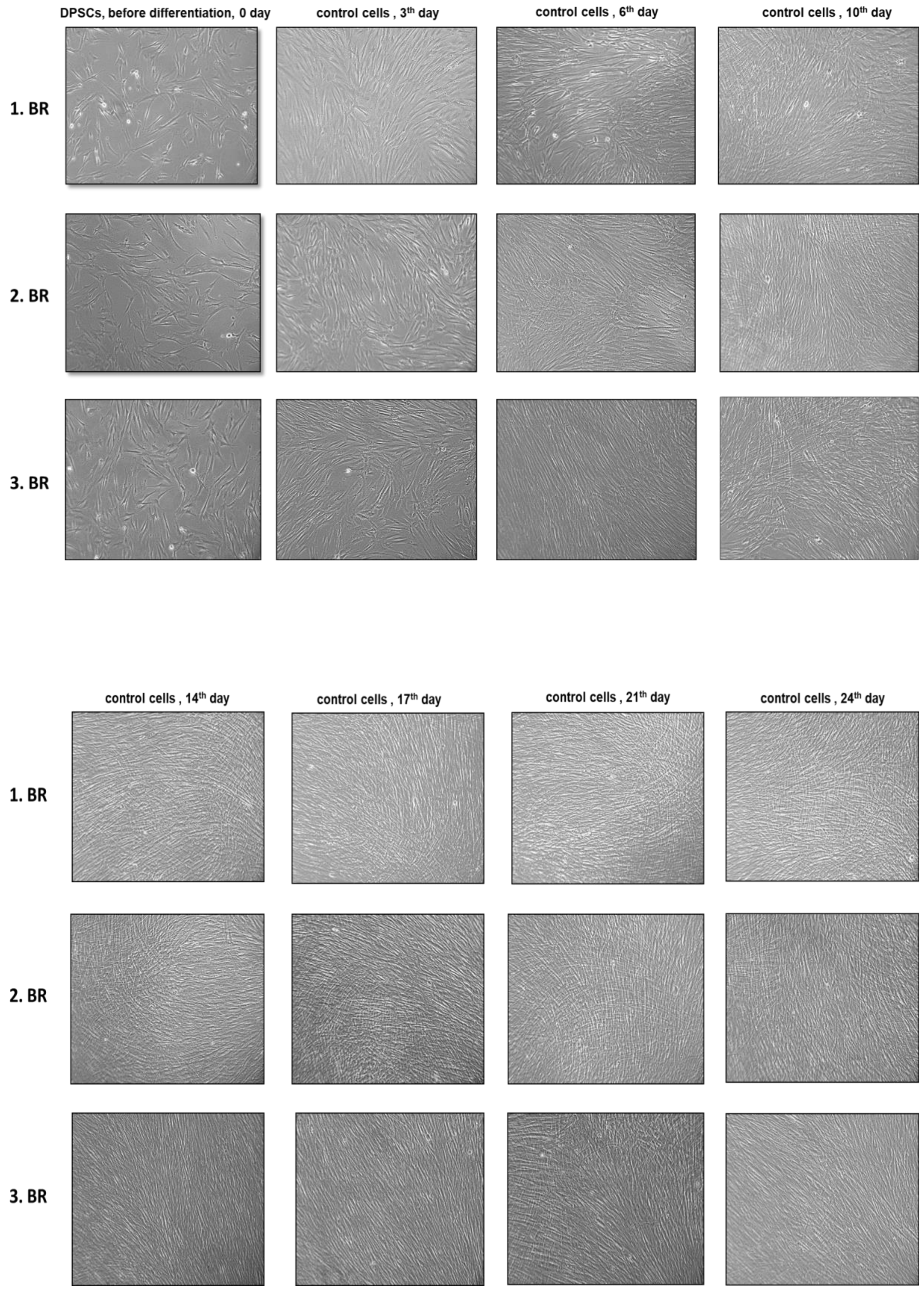

Supplementary figure S1 Morphological changes during 24 days in undifferentiated (control) (A) and differentiated cells (B). Magnification 100 x.

B

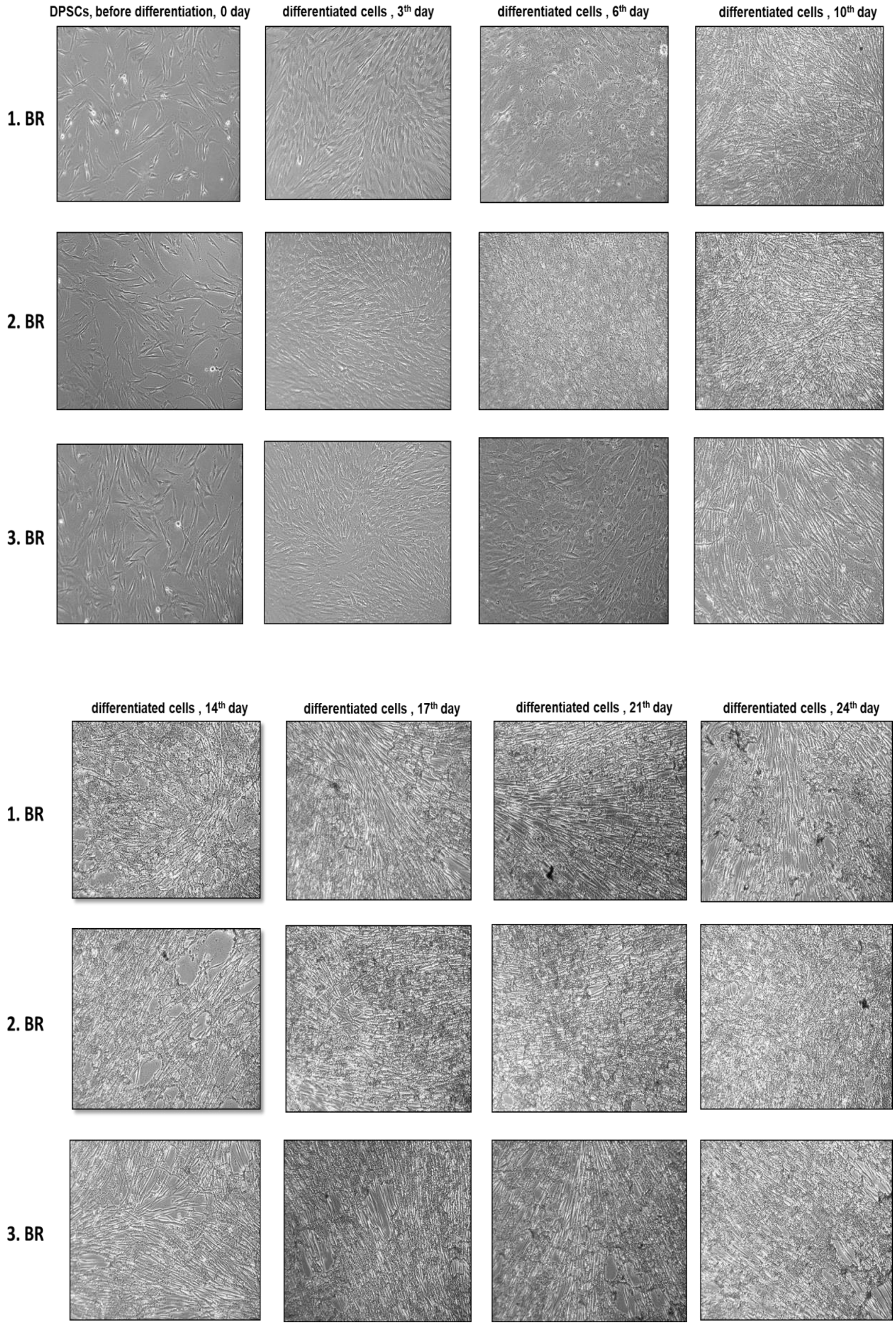

Supplementary figure S1. Morphological changes during 24 days in control (A) and osteodifferentiated cells (B). Magnification 100 x.

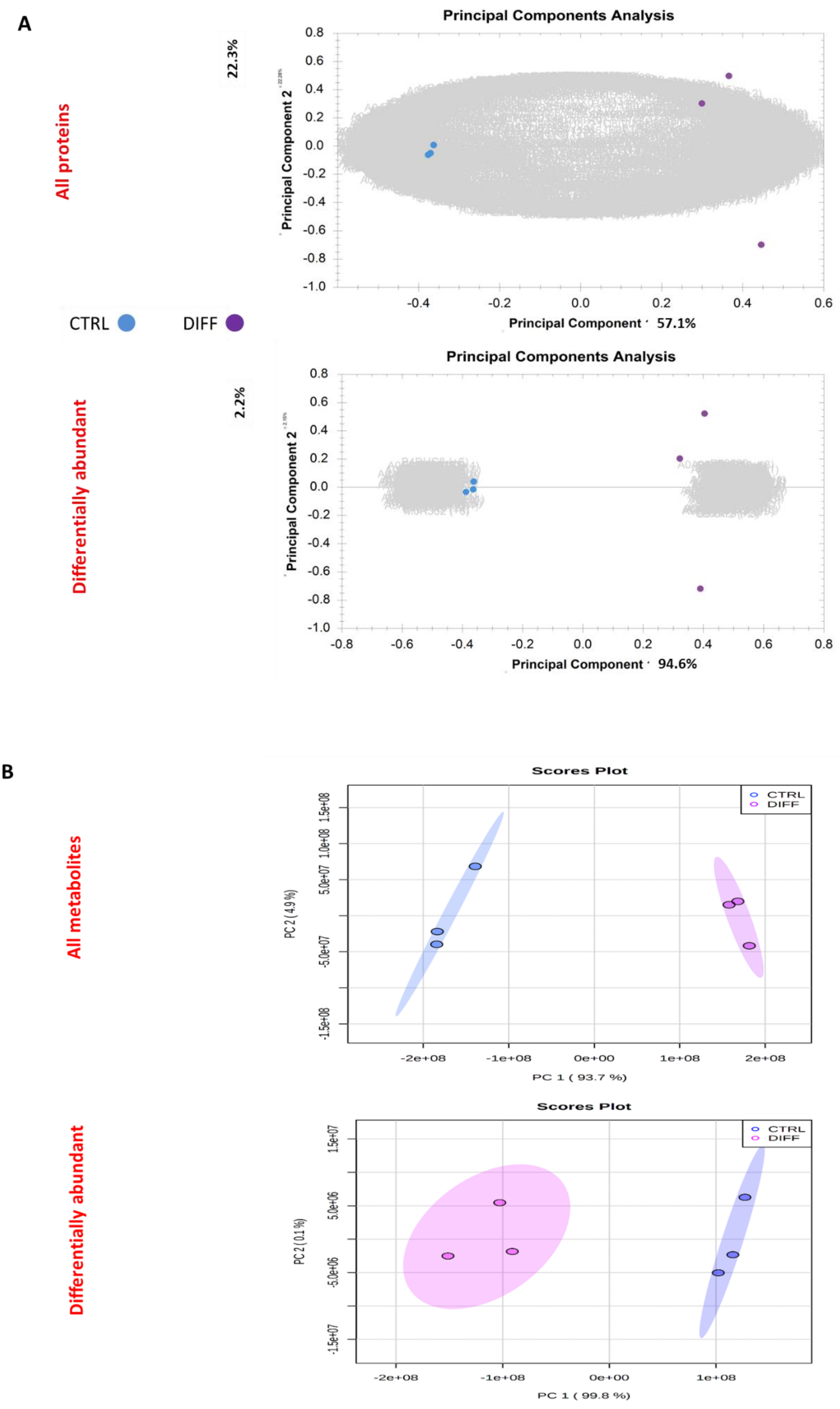

Supplementary figure S2. Principal component analysis: A—proteins, B—metabolites

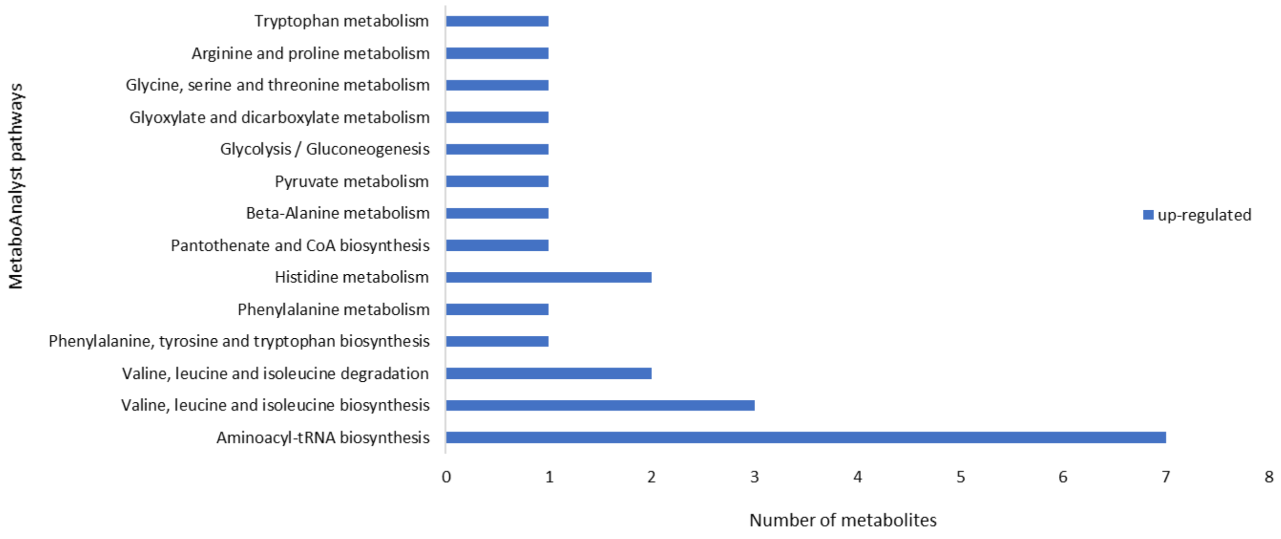

Supplementary figure S3. MetaboAnalyst pathway analysis. Column chart show functional categorization of differentially abundant metabolites into metabolic pathways.
