# Supplementary material for: Comparative Proteomic and Metabolomic Analysis of Human Osteoblasts, Differentiated from Dental Pulp Stem Cells, Hinted Crucial Signaling Pathways Promoting Osteogenesis
Source: Int J Mol Sci. 2021 Jul 24;22(15):7908. doi: 10.3390/ijms22157908 (PMC8347416; doi:10.3390/ijms22157908)
Supplement: Supplementary file 1 [file ijms-22-07908-s001.zip › Appendix C, Original images for blots.pdf]

Comparative proteomic and metabolomic analysis of human osteoblasts, differentiated from dental pulp stem cells, hinted crucial signaling pathways promoting osteogenesis

Slavomíra Nováková[1*#], Maksym Danchenko[2#], Terézia Okajčeková[1], Eva Baranovičová[1], Andrej Kováč[3], Marián Grendár[1], Gábor Beke[4], Janka Pálešová[1], Ján Strnádel[1], Mária Janíčková[5], Erika Halašová[1,6] and Henrieta Škovierová[1*]

[1]Biomedical Centre Martin, Jessenius Faculty of Medicine in Martin, Comenius University in Bratislava (JFM CU), Malá Hora 4C; 036 01 Martin, Slovak Republic

[2]Plant Science and Biodiversity Center, Slovak Academy of Sciences, Dúbravská cesta 9, 845 23 Bratislava, Slovak Republic

[3]Institute of Neuroimmunology, Slovak Academy of Sciences; Dúbravská cesta 9, 845 10 Bratislava, Slovak Republic

[4]Institute of Molecular Biology, Slovak Academy of Sciences; Dúbravská cesta 21, 845 51 Bratislava, Slovak Republic

[5]Department of Stomatology and Maxillofacial Surgery, University Hospital in Martin and JFM CU, Kollárova 2, 036 01 Martin, Slovak Republic

[6]Department of Medical Biology, Jessenius Faculty of Medicine in Martin, Comenius University in Bratislava (JFM CU), Malá Hora 4, 036 01 Martin, Slovak Republic

*Correspondence: henrieta.skovierova@uniba.sk; Tel.: +421 43 2633 904 (H.S.)
slavomira.novakova@uniba.sk; Tel.: +421 43 2633 904 (S.N.)
# Both authors contributed equally to this work

Table of content

Original Images for Blots

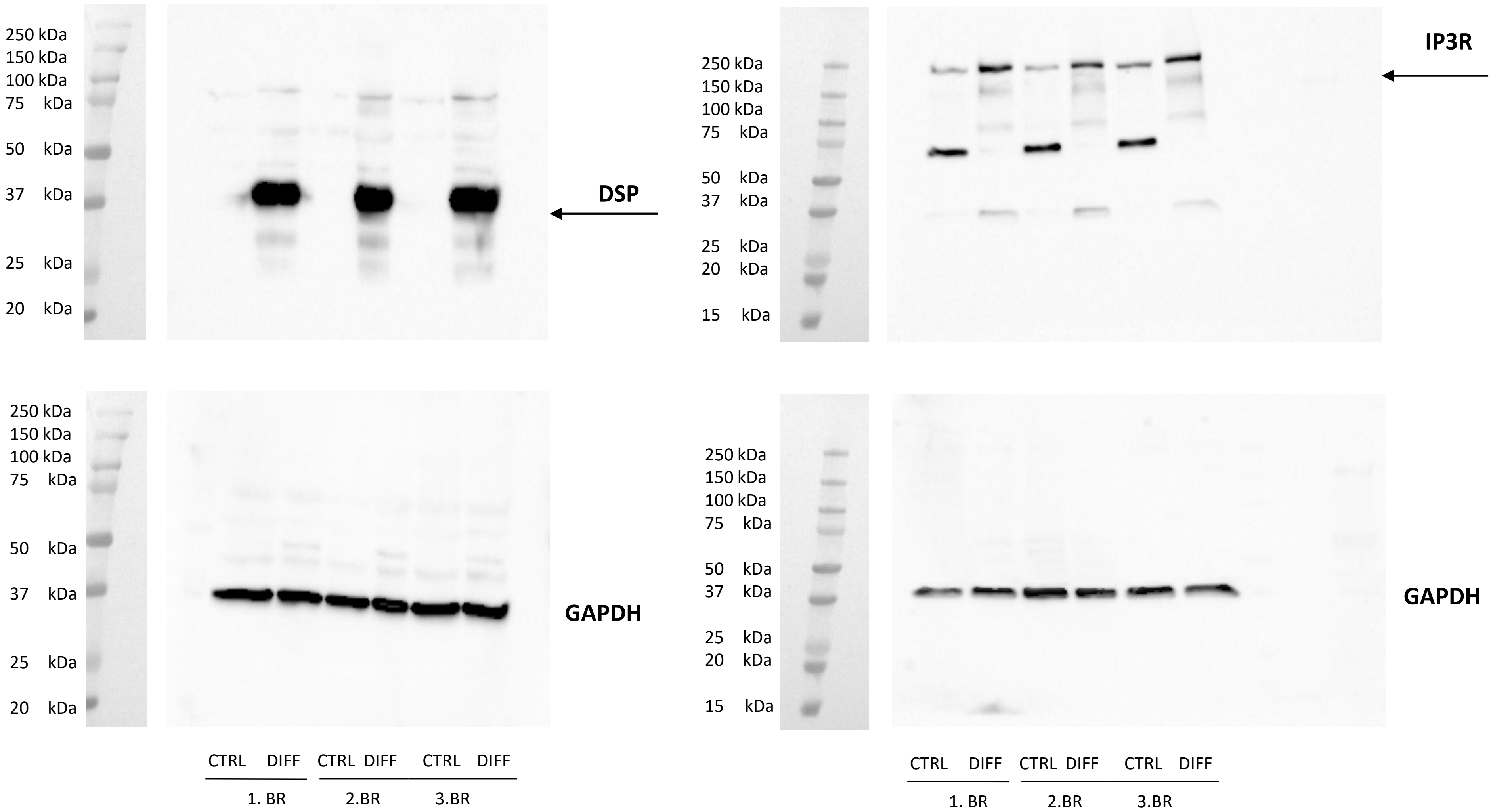
250 kDa
150 kDa
100 kDa
75 kDa
50 kDa
37 kDa
25 kDa
20 kDa
15 kDa
DSP
IP3R
GAPDH
GAPDH
CTRL DIFF CTRL DIFF CTRL DIFF
1. BR
2.BR
3.BR
CTRL DIFF CTRL DIFF CTRL DIFF
1. BR
2.BR
3.BR

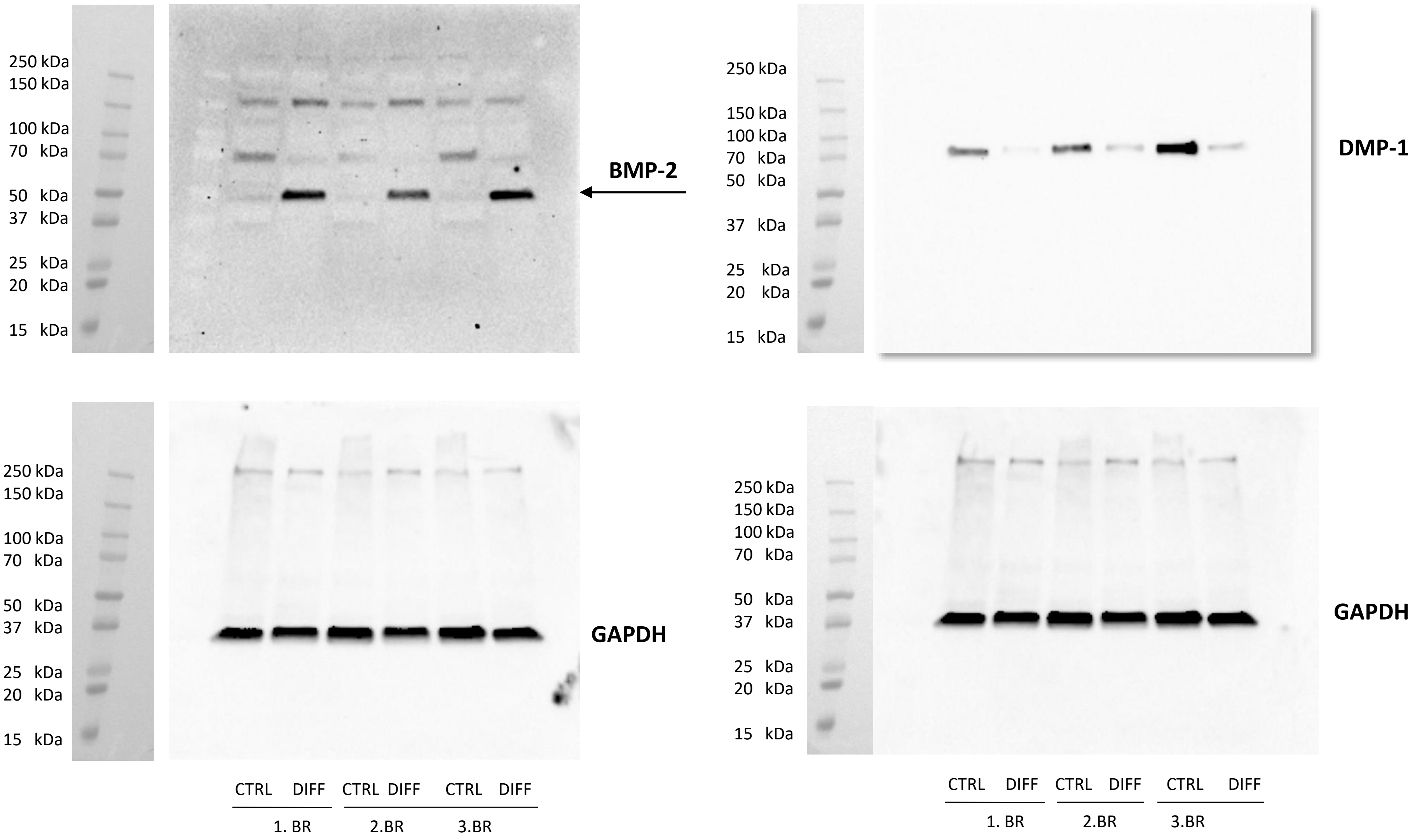

250 kDa
150 kDa
100 kDa
70 kDa
50 kDa
37 kDa
25 kDa
20 kDa
15 kDa
BMP-2
DMP-1
GAPDH
GAPDH
CTRL DIFF CTRL DIFF CTRL DIFF
1. BR
2.BR
3.BR

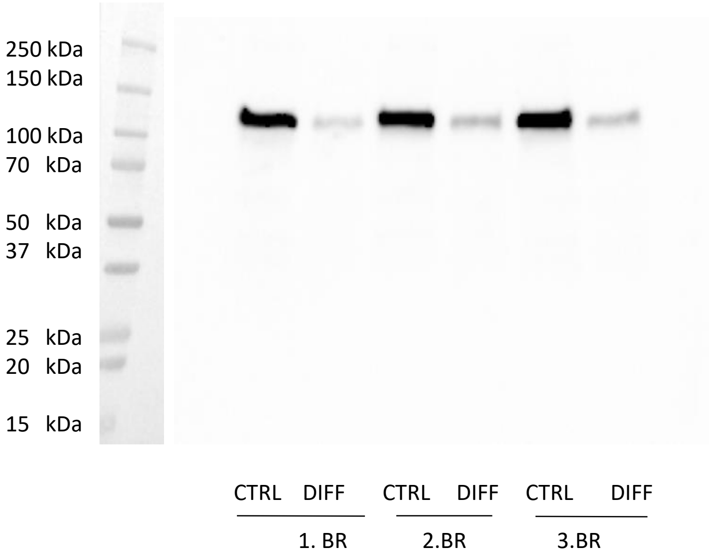
250 kDa
150 kDa
100 kDa
70 kDa
50 kDa
37 kDa
25 kDa
20 kDa
15 kDa
CTRL DIFF CTRL DIFF CTRL DIFF
1. BR
2.BR
3.BR

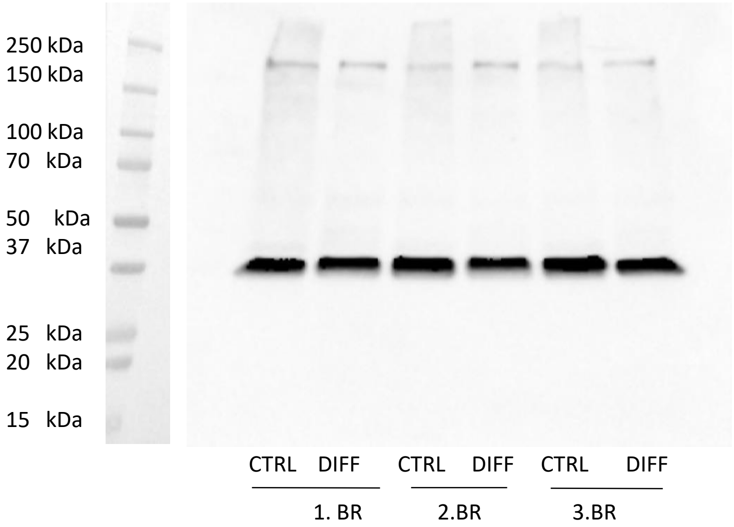
250 kDa
150 kDa
100 kDa
70 kDa
50 kDa
37 kDa
25 kDa
20 kDa
15 kDa
CTRL DIFF CTRL DIFF CTRL DIFF
1. BR
2.BR
3.BR
